# Investigation of the pathogenic variants induced Sjogren’s syndrome in Turkish population

**DOI:** 10.1515/med-2025-1294

**Published:** 2025-10-06

**Authors:** Ulku Terzi, Ilker Ates, Abdulsamet Erden, Sinan Suzen, Lalu Muhammad Irham, Serkan Yilmaz

**Affiliations:** Department of Pharmaceutical Toxicology, Faculty of Pharmacy, Ankara University, Ankara, Turkey; Department of Immunology and Rheumatology, Faculty of Medicine, Gazi University, Ankara, Turkey; Faculty of Pharmacy, Univeristas Ahmad Dahlan, Yogyakarta, Indonesia; Department of Midwifery, Faculty of Nursing, Ankara University, Hacettepe Dist. Plevne Str. No:7 06230 Altındağ, Ankara, Turkey

**Keywords:** Sjögren’s syndrome, gene polymorphism, autoimmune diseases, variants

## Abstract

**Background:**

Sjögren’s syndrome (SS) is a chronic autoimmune disorder primarily affecting the exocrine glands, leading to symptoms such as dry eyes and mouth, joint pain, fever, and neurological complications. The etiology of SS involves a complex interplay of hormonal, immune, environmental, and genetic factors. Previous studies have identified associations between SS susceptibility and polymorphisms in genes such as HLA-II, STAT4, BAFF, and TNIP1. However, these associations have not been explored in the Turkish population.

**Objective:**

This study aimed to investigate the association between four specific single nucleotide polymorphisms (SNPs) – rs1130380, rs7574865, rs9514828, and rs17728338 – and susceptibility to SS in a Turkish cohort.

**Methods:**

A total of 115 SS patients and 40 healthy controls were recruited from Turkey. Genomic DNA was extracted, and genotyping of the four selected SNPs was performed using the polymerase chain reaction – restriction fragment length polymorphism method. Genotypic and allelic distributions were compared between the patient and control groups.

**Results:**

Significant associations were found between the analyzed polymorphisms and SS susceptibility. Additionally, allele frequency comparisons with global datasets revealed that the risk alleles occur at higher frequencies in the Turkish population compared to European, American, and Asian populations, indicating a potential population-specific genetic predisposition.

**Conclusion:**

These findings suggest that the SNPs rs1130380, rs7574865, rs9514828, and rs17728338 may contribute to SS susceptibility in the Turkish population. This preliminary evidence supports the need for larger, population-based studies to further elucidate the genetic underpinnings of SS in Turkey.

## 1 Introduction

Sjögren’s syndrome (SS) is a chronic autoimmune disease of unidentified etiology including exocrine glands. SS patients show dry eyes and mouth, joint pain, fever, and neurological symptoms [1]. Yet uncertain interplay of genetic, epigenetic, and environmental procedures accounts for the initiation, perpetuation, and sustainability of the autoimmune inflammatory reply toward the influenced epithelium [2]. SS is frequently named “autoimmune epithelitis” [3] since epithelial cells play a primary function in this disorder as both targets and initiators of the autoimmune process [4]. The epithelial cells create proinflammatory cytokines, which in turn guides to defective function of the salivary glands [5].

The SS can be disunited into primary SS (pSS) and secondary SS (sSS). A lack of disorders other than rheumatologic disease represents pSS [6,7], whereas sSS arises secondary to autoimmune diseases involving systemic sclerosis, systemic lupus erythematosus (SLE), or rheumatoid arthritis (RA). The moderate incidence of SS is 6.0 per 100,000 people and is ten-fold higher in women corresponded to men. The incidence of SS raises with age; the highest incidence in women happens at 55–64 years and in men at 65–74 years [8]. Although country-specific incidence data for SS in Turkey remain limited, available clinical experience and regional reports suggest the disease follows a similar demographic pattern, with a predominance in middle-aged women and frequent presentation of classic sicca symptoms (dry eyes and mouth), arthralgia, and less commonly, extraglandular manifestations. Multiple investigators have broadly examined the reasons underlying SS and have discovered multifactorial pathogenesis of SS such as genetic, environmental, neuroendocrine, and immune (related to immune cells and cytokines) factors. The pathogenesis of SS has not been completely clarified, and there stay problems in the clinical diagnosis of SS. Genetic preconception is a condition (HLA-DRB1*03: 01, DQA1*05: 01, DQB1*02: 01 [71,77], X chromosomes), but epigenetic changes [9] are also needed for the evolution of this autoimmune disorder. An improved risk for connective tissue illnesses (such as SLE and systemic scleroderma [SS]) or other autoimmune disorders has been exhibited in SS patients’ families [11].

Other elements such as immune dysregulation, hormones, and environmental impacts (among other factors, stress [12], infections [13], drugs [14], vaccines [15], or silicone breast implants [16]) are currently being conferred to guide the misdirected activation of the innate and adaptive immune system. This triggers type 1 and type 2 interferon signaling cascades that promote B cells’ proliferation. Genetic susceptibility plays an essential role in SS pathogenesis. Actual data appeared in the last years ensuring that the essential players in SS pathogenesis are the continuous activation of type I interferon (IFN-I) system concurrently with autoreactive B and T cells and disease-associated autoantibodies, therefore, showing attractive targets for individualized therapeutic strategies in SS [17]. Earlier studies utilized SS genome-wide association studies (GWAS) to specify *HLA-II, BAFF, IRF5-TNPO3, STAT4, IL12A, FAM167A-BLK, DDX6-CXCR5*, and *TNIP1* as risk locations, IRF5 and *STAT4* as susceptibility genes for SS [77].

Human leucocyte antigen (HLA) complex is positioned on the short arm of chromosome 6 [18–20]. HLA antigens are expressed on many cell surfaces and have a primary role in the acclaim of antigenic stimulants, stimulation of the immune system, and regulation of cellular and humoral immunity [19]. HLA complexes are categorized into three classes as Class I, Class II, and Class III [20]. HLA Class II proteins have the most hereditable susceptibility to autoimmune diseases such as SS. HLA-DQB1 is the beta 1 subunit of the HLA-DQ surface receptor, in the human major histocompatibility complex (MHC), part of immune regulation. It is linked to immune conditions [21]. This gene is found to be associated with several diseases; DQB1*0201 and DQB1*0302 are high risk, particularly together in type I diabetes, DQB1*06:02 form has been strongly linked with narcolepsy [22–24]. The highest risk in celiac disease is DQB1*02 form with the HLA-DQ2.5 heterodimer (HLA-DQA1*05-DQB1*02) [25], DQB1*0501 is associated with scleroderma in Chinese population [26], DQB1*03 confers susceptibility to hepatitis C in Japanese population [27] and DQB1*0202 is possibly connected to podoconiosis [28,29]. Although it has been shown that the HLA-DQB1 gene is associated with SS, there is no scientific data on the effect of the HLA-DQB1*03 rs1130380 polymorphism on the development of the disease.

The signal transducer and activator of transcription 4 (STAT4) is a partner of the STAT family and localizes to the cytoplasm. STAT4 is phosphorylated after a variety of cytokines [30] attach to the membrane and then dimerized STAT4 translocates to the nucleus to regulate gene expression. STAT4 plays a vital function in a wide variety of cells and the pathogenesis of diverse human disorders, particularly multiple kinds of autoimmune and inflammatory illnesses, through activation by various cytokines via the Janus kinase (JAK)-STAT signaling path [31]. Various combinations of STAT4 are induced by a variety of cytokines. Cytokines play essential role in a broad spectrum of biological processes, particularly in inflammation and immune response, and are key mediators of the toxic and pathogenic effects seen in humans [32] such as interleukin (IL)12, IFN-I, IL23, IL2, IL27, and IL35.

B cell-activating factor (BAFF) is a partner of the TNF superfamily that regulates immune responses. BAFF is a cytokine with influential impacts on the development and selecting of B cells [33]. It is expressed as membrane-bound BAFF and soluble protein BAFF [33,36–39]. Sjöstrand et al. [40] uncovered raised BAFF expression in the immune cells of pSS patients, mainly neutrophils. In addition, they specified an approvingly preserved IFN-stimulated response element (ISRE) area close to the BAFF gene promoter, which was functionally confirmed. The association of BAFF polymorphisms in the development of further autoimmune disorders has been revealed theretofore [34,41–49]. BAFF rs9514828 polymorphism is found to be linked to several inflammatory and autoimmune diseases via several scientific studies [48,50–53].

Tumor necrosis factor-alpha inducible protein 3 (TNFAIP3) interacting protein 1 (TNIP1) encoded by the TNIP1 gene is a vital signaling protein in the NF-κB path. It operates together with the TNFAIP3 protein to repress NF-κB activation. The association of TNIP1 gene polymorphism with many autoimmune diseases such as systemic sclerosis, RA, psoriasis, and SLE was demonstrated [54–59]. TNIP1 rs17728338 polymorphism is revealed to be associated with some inflammatory and autoimmune diseases through scientific studies [60–63].

Although many variants have been identified which were associated with SJS development, however the variants (rs1130380, rs7574865, rs9514828, and rs17728338) have not been investigated in Turkish population. Thus, the purpose of this study was to investigate whether the polymorphisms (HLA-DQB1*03 rs1130380, *STAT4* rs7574865, *BAFF* rs9514828, and *TINIP1* rs17728338) is associated with SS development in a group of Turkish patients.

## 2 Materials and methods

### 2.1 Study population

**Figure 1:**
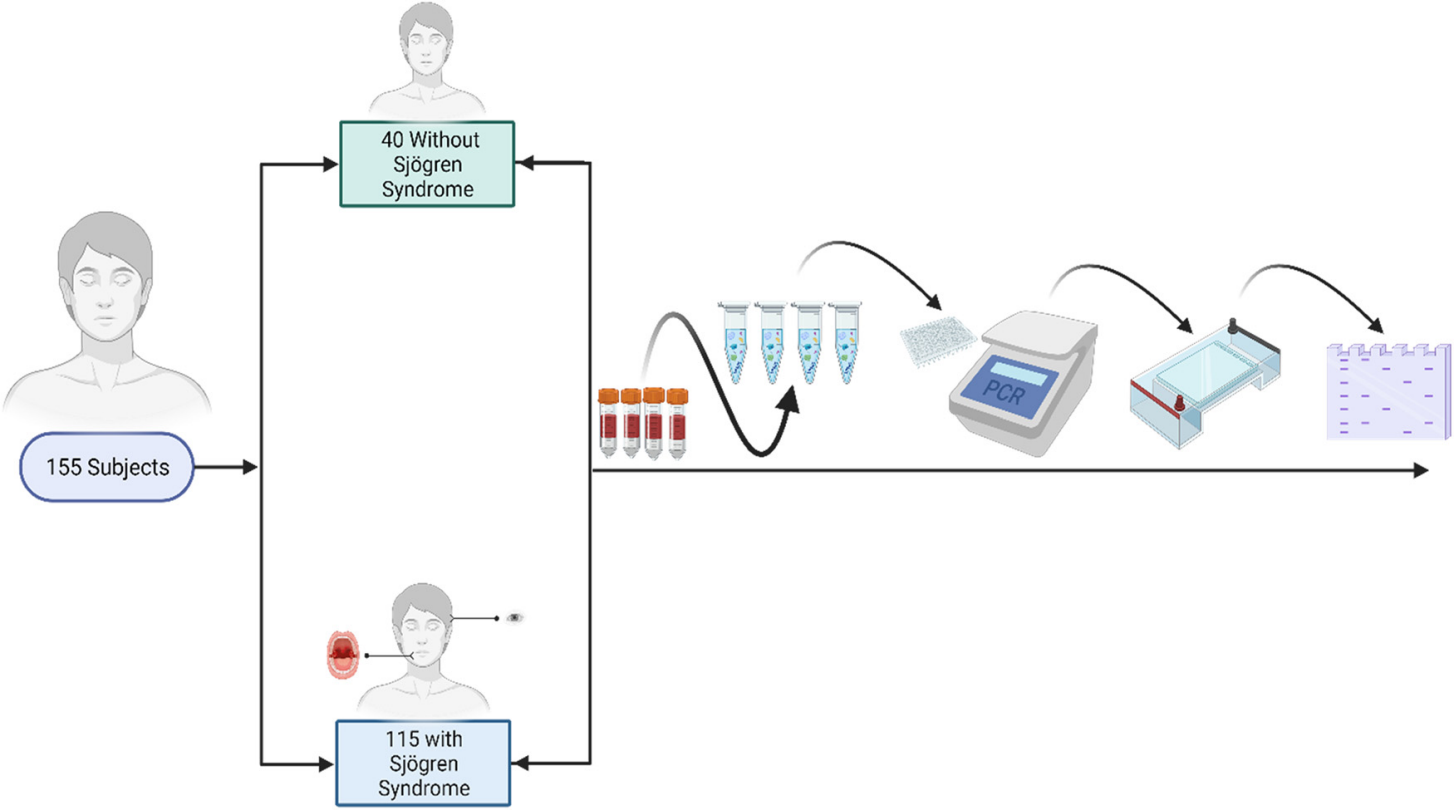
This study group consisted 40 healthy subjects and 115 patients with age range of 18–75 with SS who were attending the Rheumatology Clinic at the Ankara Bilkent City Hospital.

This study group consisted 40 healthy subjects and 115 patients with age range of 18–75 with SS who were attending the Rheumatology Clinic at the Ankara Bilkent City Hospital (Figure 1). SS was diagnosed according to the criteria set out by American College of Rheumatology/European League Against Rheumatism classification criteria for pSS [7]. Each subject responded a brief questionnaire that gives information about previous medical history, smoking, and lifestyle. Informed consent was obtained from all subjects in accordance with the Helsinki Declaration of the World Medical Association [64]. We have had approval of the Ankara University Faculty of Medicine Ethic Committee for this research and possess informed consent of each subject participating in the study. The power analysis has been performed for choosing the number of control and patient group.

### 2.2 Blood samples collection

The 5 mL blood samples of the individuals of the control and patient groups participating in the study were taken under the supervision of a doctor at the Ankara Bilkent City Hospital Rheumatology Clinic. Blood samples were taken into heparinized tubes and then transferred to Ankara University Faculty of Pharmacy, Department of Pharmaceutical Toxicology Research Laboratory with cold chain without wasting any time, where all experiments were performed. DNA isolation method, which is the preliminary step of genotyping experiments, was applied to blood samples and DNA samplings of each individual in the experimental groups were isolated. These DNA samplings were stored at −20°C until genotyping experiments were performed. Genotyping experiments were performed using the polymerase chain reaction–restriction fragment length polymorphism (PCR–RFLP) method.

### 2.3 DNA isolation and DNA quantification

The Wizard Genomic DNA Purification kit, produced by Promega, was used for the isolation of each individuals’ DNA sample.

The purity of the DNA samples we obtained was determined by dividing the absorbance value read at 260 nm in the spectrophotometer by the absorbance value read at 280 nm. Accordingly, it was determined that DNA samples with values between 1.7 and 2.0 were isolated with sufficient purity.

### 2.4 Genotyping experiments

#### 2.4.1 Determination of HLA-DQB1*03 rs1130380 gene polymorphism

The HLA-DQB1*03 rs1130380 encoded polymorphism was detected according to the method of Soetjipto et al. [10]. HLA-DQB1*03 rs1130380 polymorphism analyses were performed using PCR and RFLP methods.

Total reaction mix of 50 µL contains 5 µL 10× PCR buffer, 100 mM MgCl_2_, 100 µmol dNTP mix, 77 pmol forward primer DQBS43 (5′-TGC TAC TTC ACC A(C/T) GGG-3′) and reverse primer DQBA249 (5′-GTA GTT GTG TCT GCA (C/T)A C-3′), 5,000 U Taq DNA polymerase, and 50 ng DNA. For amplification, 35 cycles were performed using the following steps: denaturation at 94°C for 1 min, annealing at 60°C for 2 min, and extension using Taq DNA polymerase at 72°C for 3 min, with the final step after 35 cycles at 72°C for 3–10 min.

Following the PCR reaction, the RFLP method was applied by restriction with MspI restriction enzyme overnight at 37°C. After enzyme digestion, PAGE electrophoresis was performed and three bands of 99, 72, and 36 bp showed the wild type genotype (*0301), two bands of 171 and 36 bp showed heterozygous genotype (*0303), and a single band of 207 bp showed the presence of homozygous mutant genotype (*0601).

#### 2.4.2 Determination of STAT4 rs7574865 gene polymorphism

The STAT4 rs7574865 encoded polymorphism was detected according to the method of Migita et al. [35]. STAT4 rs7574865 polymorphism analyses were performed using PCR and RFLP methods.

Total reaction mix of 50 µL contains 5 µL 10× PCR buffer, 100 mM MgCl_2_, 100 µmol dNTP mix, 100 pmol forward primer (5′-AAA GAA GTG GGA TAA AAA GAA GTT TG-3′) and reverse primer (5′-CCA CTG AAA TAA GAT AAC CAC TGT-3′), 5,000 U Taq DNA polymerase, and 50 ng DNA. Denaturation step was started with initial heating at 95°C for 5 min, followed by the addition of the polymerase and then 35 cycles of denaturing (at 95°C for 20 s), annealing (at 56°C for 30 s), and chain extension (at 72°C for 1 min), followed with a final extension step at 72°C for 3 min.

Following the PCR reaction, the RFLP method was applied by restriction with HpaI restriction enzyme overnight at 37°C. After enzyme digestion, PAGE electrophoresis was performed and a single band of 147 bp showed the wild type genotype (G allele), three bands of 147, 122, and 25 bp showed heterozygous genotype (G/T allele) and two bands of 122 and 25 bp showed the presence of homozygous mutant genotype (T allele).

#### 2.4.3 Determination of BAFF rs9514828 gene polymorphism

The BAFF rs9514828 encoded polymorphism was detected according to the method of Marin-Rosales et al. [79]. BAFF rs9514828 polymorphism analyses were performed using PCR and RFLP methods.

Total reaction mix of 30 µL contains 5 µL 10× PCR buffer, 2 mM MgCl_2_, 200 µmol dNTP mix, 25 ng forward primer (5′-TTG TAC ACC GAC CTG TTA GG-3′) and reverse primer (5′-TGG AAG TAA GTC CAG ACT GGG AAT-3′), 5,000 U Taq DNA polymerase, and 40 ng DNA. The PCR conditions were initial denaturation cycle at 94°C for 3 min, 35 cycles of denaturation for 30 s at 94°C, annealing for 20 s at 57°C, extension for 30 s at 72°C, and final extension for 1 min at 72°C.

Following the PCR reaction, the RFLP method was applied by restriction with HpaI restriction enzyme overnight at 37°C. After enzyme digestion, PAGE electrophoresis was performed and two bands of 261 and 137 bp showed the wild type genotype (C allele), three bands of 398, 261, and 137 bp showed heterozygous genotype (C/T allele), and a single band of 398 bp showed the presence of homozygous mutant genotype (T allele).

#### 2.4.4 Determination of TNIP1 rs17728338 gene polymorphism

The TNIP1 rs17728338 encoded polymorphism was detected according to the method. TNIP1 rs17728338 polymorphism analyses were performed using PCR and RFLP methods.

Total reaction mix of 20 µL contains 5 µL 1× PCR buffer, 1.5 mM MgCl_2_, 200 µmol dNTP mix, 30 ng forward primer (5′-GTA TGT TTT GCA CCT AGC ACG T-3′) and reverse primer (5′-CCA TTC GGG AGC CTT TTG CCA-3′), 1 U Taq DNA polymerase, and 20 ng DNA. Samples were denatured at 95°C for 2 min followed by 30 cycles of 94°C for 45 s, annealing for 45 s at 72°C, and ended with a final extension for 7 min at 72°C.

Following the PCR reaction, the RFLP method was applied by restriction with NcoI restriction enzyme overnight at 37°C. After enzyme digestion, PAGE electrophoresis was performed and a single band of 197 bp showed the wild type genotype (G allele), three bands of 197, 174, and 23 bp showed heterozygous genotype (G/A allele) and two bands of 174 and 23 bp showed the presence of homozygous mutant genotype (A allele).

### 2.5 Statistical analysis

Statistics analysis was performed by SPSS software version 27.0 for windows (SPSS Inc., Chicago, IL, USA). The frequencies of genotypes and alleles for all the studied polymorphisms were determined assuming co-dominant inheritance. The statistical significance of the genotype and allele frequency variables between the patients with SS and control group was evaluated by chi-square test with Yates correction for small numbers. Using the chi-square test, Hardy–Weinberg equilibrium was tested for the studied four single nucleotide polymorphism (SNPs) in patients with SS and controls. Relative risk associated with the significant genotype was estimated by the odds ratio (OR). OR with 95% confidence intervals (95% CI) was tested using a chi-square distribution, and the null hypothesis being tested is OR = 1. Allele frequencies were also evaluated. *P*-values <0.05 were considered as statistically significant.


**Informed consent:** Informed consent was obtained from all subjects participating in the study.
**Ethical approval:** The study was conducted in accordance with the Helsinki Declaration of the World Medical Association and had the approval of the Ankara University Faculty of Medicine Ethic Committee.

## 3 Results

The aim of this study is to identify the association between four variants including rs1130380 encoded the HLA-DQB1*03 gene, rs7574865 encoded *STAT4* gene, rs9514828 encoded the *BAFF* gene, and rs17728338 encoded the *TINIP1* gene and the development of the SS. This study recruited 155 subjects (40 healthy subjects and 115 patients with SS) in a Turkish population. In Table 1 the demographic data of the all subjects are shown.

**Table 1:** Demographic data of the subjects

Parameter	Control group		Patient group	
	Male	Female	Male	Female
Number of subjects	3	37	10	105
Age (mean ± SD)	53.33 ± 4.51	53.43 ± 11.92	54.40 ± 7.97	53.79 ± 10.77
Smoking status (%)				
Yes	1 (33.3%)	8 (21.6%)	2 (20.0%)	21 (20.0%)
No	2 (66.7%)	29 (78.4%)	8 (80.0%)	84 (80.0%)

Correlations between SS age and smoking were evaluated by multiple linear regressions but no significant association was obtained.

Age, smoking, gender, family history, anti-Ro/SSA seropositivity, anti-La/SSB seropositivity, RF seropositivity, ANA seropositivity, SS type (primer/seconder), and disease year were evaluated as risk factors for SS so they were statistically analyzed by multiple logistic regression analysis. Due to the data, except age and smoking all other factors have a significant relationship with SS development (family history and type of SS factors’ significance limit is <0.05; gender, disease year, and all seropositivity factors’ significance limit is <0.01). All the data related to these are shown in Table 2.

**Table 2:** Multiple logistic regression analysis for possible risk factors of SS

Risk factor	OR (95% CI)
Age	0.67 (0.23–0.91)
Smoking	1.21 (0.57–1.87)
Gender	3.09 (2.07–5.78)^b^
Family history (%)	1.48 (1.04–2.70)^a^
Anti-Ro/SSA seropositivity	3.17 (1.64–6.25)^b^
Anti-La/SSB seropositivity	2.91 (1.53–5.11)^b^
RF seropositivity	2.55 (1.49–4.27)^b^
ANA seropositivity	2.04 (1.63–3.78)^b^
Primer/seconder SS	1.61 (1.15–3.44)^a^
Disease year (*x̄*)	2.21 (1.53–4.03)^b^

^a^ *P* < 0.05.

^b^ *P* < 0.01.

The genotypic distribution of all the studied gene polymorphisms is demonstrated in Table 3. According to the outcomes, all examined variants displayed a significant relationship with the development of the disease. The HLA-DQB1*03 1/2 and 2/2 allele carriers were significantly over-represented in SS patients. The OR for SS with the HLA-DQB1*03 variant was admiringly raised (OR = 6.05, 95% CI: 2.78–13.20). The OR of the STAT4 variant was improved in SS patients about six-fold similarly with HLA-DQB1*03 variant (OR = 5.88, 95% CI: 2.69–12.86). Likewise with the others, BAFF variant was amazingly triggered in SS patient about seven-fold compared with the controls (OR = 6.71, 95% CI: 3.05–14.79). With regard to the last studied variant TNIP1, it was also raised in SS patients compared with the controls and this enhancement was significant (OR = 4.87, 95% CI: 2.26–10.50). All the impacts had a *P*-value smaller than 0.001. Genotype distributions for all the gene polymorphisms suit forecasts for Hardy–Weinberg equilibrium.

**Table 3:** Genotypic distribution of the studied genes

Region	1/1 Allele* (%)	1/2 or 2/2 Allele (%)	Total	OR* (CI)	*P*
HLA-DQB1*03 rs1130380					
Control	26 (65.0)	14 (35.0)	40	1.00	<0.001
Patient	27 (23.5)	88 (76.5)	115	6.05 (2.78–13.20)	
*STAT4 rs7574865*					
Control	27 (67.5)	13 (32.5)	40	1.00	***<*** 0.001
Patient	30 (26.1)	85 (73.9)	115	5.88 (2.69–12.86)	
*BAFF rs9514828*					
Control	24 (60.0)	16 (40.0)	40	1.00	***<*** 0.001
Patient	21 (18.3)	94 (81.7)	115	6.71 (3.05–14.79)	
*TNIP1 rs17728338*					
Control	23 (57.5)	17 (42.5)	40	1.00	***<*** 0.001
Patient	25 (21.7)	90 (78.3)	115	4.87 (2.26–10.50)	

*1 allele major variant, 2 allele minor variant.

In Table 4, allele frequencies of all the studied gene polymorphisms are given. When we evaluate the results, it is clearly seen that the allele 2 frequencies were about two folds increased in SS patients compared to the controls.

**Table 4:** Allele frequencies of the studied genes

Allele	Allele frequency			
	HLA-DQB1*03	*STAT4*	*BAFF*	*TNIP1*
Control group				
1/1	0.775	0.790	0.725	0.730
2/2	0.225	0.210	0.275	0.270
Patient group				
1/1	0.505	0.510	0.465	0.510
2/2	0.495	0.490	0.535	0.490
P*	*<*0.01	*<*0.01	*<*0.05	*<*0.05

*Comparison between the 2/2 variant of the patients with those of controls.

To the best of our knowledge, this study is the first where all these four gene polymorphisms are thought to have a relationship with SS development at the same time especially in Turkish population.

The allele frequency of each variant (rs1130380, rs7574865, rs9514828, rs17728338) was then assessed in regional populations including African, American, European, and Asian cohorts. The results of this study indicate that (rs1130380, rs7574865, rs9514828, rs17728338) upregulating variants are more common in Turkish populations than in European, American, and other Asian populations, suggesting that Turkish populations may be more susceptible to the development of SS (Table 5).

**Table 5:** Allele frequencies’ SNPs examined in this study and multiple continents

SNP	Position (hg19) (bp)	Gene	Location	Allele		Allele frequencies (N)				
				Ref	Eff*	EUR	AFR	AMR	ASN	Our
rs1130380	chr6: 32632694	*HLA-DQB1*	Missense	C	A, G, T	0	0	0	0	0.495
rs7574865	chr2: 191964633	STAT4	Intron	T	G	0.77	0.86	0.69	0.66	0.490
rs9514828	chr13: 108921373	*BAFF*	—	C	T	0.53	0.09	0.28	0.40	0.535
rs17728338	chr5: 150478318	*ANXA6*	—	C	T	0.07	0.07	0.05	0.11	0.490

EUR, European; AFR, African; AMR, American; ASN, Asian; Ref, reference, and Eff, effect allele of EUR, AFR, AMR, ASN were extracted from the HaploReg Version 4.1 (https://pubs.broadinstitute.org/mammals/haploreg).

## 4 Discussion

SS is a chronic autoimmune disease and genetic susceptibility plays a major role in SS pathogenesis. Chused et al. first demonstrated HLA genes as a risk factor for SS in 1977 [65]. The relationship between HLA-DR3 and SS was demonstrated especially in the white ethnicity. On the other side, association between SS and HLA-DR3-DQ haplotypes was revealed in various ethnic groups [66–76]. Numerous methods have been accustomed including the genetic linkage analysis, positional candidate gene analysis using microsatellite markers, and more newly the GWAS studies completed on a huge series of SS patients and controls with SNP markers [77–79].

Nevertheless, a meta-analysis pinpoints DRB1*0301, DQA1*0501, DQB1*0201, and DRB1*03 alleles as risk factors for SS, while pinpointing DQA1*0201, DQA1*0301, and DQB1*0501 alleles as preservatives [80]. Lately, a powerful connection between HLA-DRA, HLA-DQB1, and HLA-DQA1 and SS in 6p21 locus in an extensive study in Europe was documented [77]. In a Chinese research, HLA-DRB1/HLA-DQA1 in 6p21.3 locus and two separate signals linked with HLA-DPB1/COL11A2 [102]. Degeneration of autoreactive T cell toleration via the existence of unnatural antigen displays the primary role of HLAs in autoimmune diseases. The disorder association of HLA-suspected alleles is familiar in autoimmune diseases and further specific alleles and haplotypes are developed, extra alleles with simple targeting of typical autoantigens [81]. HLA Class II is linked with autoantibody production in SS, whereas anti-Ro/SSA and anti-La/SSB are particularly more elevated in HLA-DQ1/HLA-DQ2 heterozygous patients [82] but not connected to further clinical characteristics [71]. HLA-DRB1*1501-DRB1*0301 is associated with anti-anticyclic citrullinated antibodies [83]. Amino acid deviations in the hypervariable province of the HLA complex influence peptide binding and T cell display. The association of specific divergences in binding wells 7 and 9 of HLA-DRB1 with differences in depth and polarity was established in the Chinese population [75]. Although HLA Class I and HLA Class III genes were also investigated in the subsequent years, investigations concentrated on HLA Class II genes.

Multitudinous studies were performed showing the relationship between HLA alleles and SS. Different genetic polymorphisms of HLA have been associated with predisposition and/or outcome of different infectious diseases such as hepatitis B virus, hepatitis C virus, chikungunya, Chagas, dengue, influenza A, and tuberculosis [84–88]. A meta-analysis performed by Cruz-Tapias et al. [80] pinpointed that some of HLA alleles such as DRB1*0301, DQA1*0501, DQB1*0201, and DRB1*03 were risk factors for SS and the others including DQA1*0201, DQA1*0301, and DQB1*0501 as preservatives. Recently, a strong association between HLA-DRA, HLA-DQB1, and HLA-DQA1 and SS in 6p21 locus in a comprehensive study in Europe was reported [77]. HLA-DQB1 is the beta 1 subunit of the HLA-DQ surface receptor, in the MHC, part of immune regulation. It is linked to immune disorders [21]. This gene is found to be linked with some disorders such as type I diabetes, narcolepsy, celiac disease, and scleroderma. Although it has been documented that the HLA-DQB1 gene is associated with SS, there is no scientific data on the impact of the HLA-DQB1*03 rs1130380 polymorphism on the development of the disease. From this point of view, our results are very valuable in terms of evaluating the possible effect of HLA-DQB1 gene variation on SS. Due to our results, HLA-DQB1*03 rs1130380 variation had a significant relationship with the development of SS about six-fold compared with controls (OR = 6.05, 95% CI: 2.78–13.20).

STAT4 is a member of the STAT family and it has a crucial position in a broad variety of cells and the pathogenesis of various human diseases, particularly multiple types of autoimmune and inflammatory disorders, via activation by different cytokines via the JAK-STAT signaling path [31]. STAT4 is a critical transcription factor for the transmission of IL-12, IL-23, and IFN-I-mediated signals implicated in Th1 and Th17 differentiation, activation of monocytes, and INFγ production [89–91]. STAT4 haplotypes have been suggested to be a risk factor for the development of SLE and RA in the Caucasians and its connection with SS [92]. STAT4 polymorphism was investigated in different ethnic groups in other loci such as rs7574865 [93] and rs7582694 [94]. In these three studies, it was pointed that rs7582694 polymorphism posed a risk for SS. rs7574865 of the STAT4 gene has been reported in a massive study of Swedes to be connected to both lupus (SLE) and RA [95]. RA risk associated with rs7574865(T) allele was also discovered in studies of 923 Spanish, 273 Swedish, and 876 Dutch patients [96]. The rs7574865(T) allele is linked with an increased risk for type-1 diabetes established by a study of Greek patients [97]. In research including 2,776 Spanish subjects, the rs7574865(T) allele was observed to be associated with RA, Crohn’s disease, ulcerative colitis, and type-1 diabetes, but not with multiple sclerosis [98]. In a study of 124 Caucasian patients with pSS, the rs7574865(T) allele was linked with a moderately increased risk for the disease [92]. Similarly, we found this variant to be associated with approximately a six-fold increased risk of SS development compared to controls (OR = 5.88, 95% CI: 2.69–12.86). These findings align with broader evidence that the STAT4 rs7574865 T allele confers susceptibility to multiple autoimmune diseases, including SS, supporting its role as a genetic risk factor without overstating the magnitude of effect.

BAFF is a member of the TNF superfamily which regulates immune responses. Sjöstrand et al. [40] discovered extended BAFF expression in the immune cells of pSS patients, especially neutrophils. They also established an admiringly preserved ISRE area close to the BAFF gene promoter, which was functionally confirmed. The association of BAFF polymorphisms in the development of additional autoimmune diseases has been displayed previously [37,41,43–49]. BAFF rs9514828 polymorphism is found to be connected with numerous autoimmune and inflammatory disorders such as SS, lymphoma, leukemia, haemophilia, and periodontitis through some scientific investigations [48,50–53]. In an investigation by Nezos et al. [48], it was found that high risk pSS group was characterized by higher frequency of the minor T allele of the rs9514828 BAFF polymorphism compared to healthy controls. Our findings are also in line with this study in which we found that in SS patients the minor variant had approximately seven-fold risk compared to the controls (OR = 6.71, 95% CI: 3.05–14.79).

Recent advances in artificial intelligence (AI) have revolutionized biomedical research and clinical practice by enabling integration and interpretation of complex multidimensional data. For example, AlphaFold accurately predicts protein structures from sequences, accelerating drug discovery [99], while AI models analyzing clinical imaging improve personalized diagnosis and treatment [100]. Our validated genetic dataset offers a rich resource for training AI models aimed at early SS risk prediction and diagnostic classification, underpinning future multimodal “genetic–clinical–imaging” AI frameworks. These approaches promise transformative improvements in population-specific precision medicine, particularly for ethnically distinct groups such as the Turkish cohort studied here.

In addition to elucidating genetic susceptibility, this study highlights the therapeutic potential of the identified genes as drug targets for SS, opening avenues for drug repurposing strategies. Although HLA-DQB1 is primarily recognized as a pharmacogenetic marker guiding drug choice rather than a direct target, the other genes have more direct therapeutic relevance. STAT4 functions within the JAK-STAT signaling pathway, and JAK inhibitors, which are approved for autoimmune diseases such as RA and psoriatic arthritis, are actively being explored in clinical trials for SS treatment. BAFF, a critical regulator of B cell survival and activation, is an established drug target; belimumab (FDA-approved for SLE) and ianalumab (in Phase III trials) target BAFF signaling and have shown promising efficacy in SS, with ianalumab demonstrating significant disease activity reduction in pivotal Phase III studies [101]. TNIP1 (ANXA6) is currently an investigational target with no approved drugs yet, but its role in NF-κB pathway regulation makes it a potential candidate for future therapeutic development. Thus, our genetic findings not only broaden understanding of underlying disease mechanisms but also underline key molecular targets with repurposing potential, facilitating precision medicine approaches in SS.

Importantly, rs9514828 is located within the promoter region of the BAFF gene and is believed to influence gene expression by altering transcription factor binding affinity. The minor allele at this locus may enhance promoter activity, resulting in elevated BAFF levels. This increase in BAFF expression can lead to exaggerated B cell activation, a key event in SS pathogenesis. Our data support this mechanism, linking the rs9514828 variant’s significant association with SS susceptibility in the Turkish population to a possible functional upregulation of BAFF expression.

TNIP1 is encoded by the TNIP1 gene which is a critical signaling protein in the NF-κB path. It functions with the TNFAIP3 protein together to suppress NF-κB activation. The connection of TNIP1 gene polymorphism with multiple autoimmune disorders such as systemic sclerosis, RA, psoriasis, and SLE was demonstrated [54–59]. TNIP1 rs17728338 polymorphism is revealed to be linked to several inflammatory and autoimmune diseases including psoriatic arthritis and psoriasis via scientific investigations [60–63]. There are limited studies related to the TNIP1 rs17728338 polymorphism and SS. Nordmark et al. [59] figured out that polymorphisms in TNIP1 are related to antibody-positive pSS. We also found that minor variant of the TNIP1 polymorphism was a risk factor in SS patients with about five-fold compared to the control group (OR = 4.87, 95%CI: 2.26–10.50).

Mechanistically, TNIP1 functions as a key negative regulator of the NF-κB signaling pathway by working in concert with the TNFAIP3 (A20) protein to repress NF-κB activation. The rs17728338 variant may affect TNIP1 expression or its protein function, potentially attenuating its inhibitory effect on NF-κB. This could result in prolonged or enhanced NF-κB signaling, fostering a pro-inflammatory environment contributing to SS pathogenesis. Our finding of a significant association of rs17728338 minor allele with SS supports the variant’s possible role in dysregulating immune responses through the NF-κB pathway in the Turkish population.

We acknowledge that this study calls for further validation with larger cohorts and other populations to enhance the generalizability and statistical power of the findings. In particular, the relatively small control group size represents a limitation that may affect the robustness of our conclusions. Nevertheless, our data provide valuable evidence that polymorphisms rs1130380, rs7574865, rs9514828, and rs17728338 are significantly associated with SS susceptibility in the Turkish population, likely through impacting key immunoregulatory pathways involving HLA-DQB1, STAT4, BAFF, and TNIP1. Future studies with expanded sample sizes and diverse populations are essential to confirm and extend these insights.

## 5 Conclusion

This study identified significant associations between four SNPs (rs1130380, rs7574865, rs9514828, rs17728338) and SS in the Turkish population, supporting their potential role as genetic risk factors. While these polymorphisms may contribute to SS susceptibility, additional studies with larger cohorts and functional analyses are required to validate their utility as biomarkers for risk assessment.
